# Editorial: Women in neuroscience

**DOI:** 10.3389/fnint.2022.1032506

**Published:** 2022-09-20

**Authors:** Arianna Maffei, Michela Chiappalone, Liana Fattore, Elizabeth B. Torres, Marie-Ève Tremblay, Corette J. Wierenga

**Affiliations:** ^1^Department of Neurobiology and Behavior, Stony Brook University, Stony Brook, NY, United States; ^2^Department of Informatics, Bioengineering, Robotics and Systems Engineering, University of Genova, Genova, Italy; ^3^Neuroscience Institute, National Research Council, Monserrato, Italy; ^4^Department of Psychology, Computational Biomedicine Imaging and Modelling Center, Computer Science Department and Rutgers University Center for Cognitive Science, Rutgers University, Piscataway, NJ, United States; ^5^Division of Medical Sciences, University of Victoria, Victoria, BC, Canada; ^6^Biology Department, Faculty of Science, Utrecht University, Utrecht, Netherlands

**Keywords:** neuroscience, neurophysiology, cognitive science, neuroengineering, neurodegeneration, neuromodulation

The “Matilda effect” is an expression coined in 1993 by Margaret Rossiter, a prominent science historian, to describe the faint recognition of the contribution of women to the scientific enterprise. The expression derived from the realization that just like the work of Matilda Gage, a suffragist who also wrote about women in science, the discoveries and inventions of many women scientists had been forgotten over the course of history. Indeed, women's contributions to science have been often misappropriated, forgotten or, in some cases, even actively removed from the records. This resulted in a misplaced historic assumption that women lack the intellectual ability and interest for scientific disciplines, and left younger generations of women with very few role models to look up to. Over the past few years, the awareness of this lack of recognition has increased and, despite encountering some resistance, active efforts have been made to make science a more inclusive enterprise. Neuroscience is a multidisciplinary field that encompasses all scientific disciplines including biology, psychology, cognitive sciences, physics, engineering, and mathematics. While women to this day represent a minority of neuroscience faculty, they contribute to all aspects of the field. The goal of the *Women in neuroscience* Research Topic is to oppose the “Matilda effect” by bringing together excellent research by women, or in collaboration with women. The Research Topic brings together 33 articles in which the first or last author are women. The formats include mini-reviews and reviews of the exceptional work done by past and present women neuroscientists, an opinion article, perspectives and specific Research Topic reviews highlighting scholarship and innovative frameworks, and original research articles that push the field forward.

Malerba describes the inspiration of working with Dr. Levi-Montalcini by focusing on the last years of research of one of the few women Nobel Laureates. Heiden et al., Yevoo and Maffei, Gonzalez Osorio et al., and Quattrocolo et al. highlight some of the excellent and impactful work by female researchers in neuroscience and medicine with the goal of emphasizing their scientific discoveries and providing role models for future scientists.

On the technological side, the work of women scientists demonstrates creativity and long-term vision. Le Bars et al. propose an innovative Brain Computer Interface (BCI) paradigm inspired by the ideomotor principle, while Shim et al. present a Machine Learning (ML) method to classify electroencephalographic oscillations, which outperforms current neurophysiological-based approaches to diagnose Post Traumatic Stress Disorder (PTSD). By exploiting fNIRS (functional near-infrared spectroscopy), Zhang M. et al. compared hemodynamic responses to low vs. high Informational Masking speech. Focusing on the human-machine teaming, Hopko and Mehta discuss the neural correlates of trust in automation and Douibi et al. envisage the (not so far) large-scale deployment of BCIs in the Industry 4.0, widening their possible applications in education, entertainment and aviation.

Numerous groups also investigated sex differences and the role of sex hormones in brain functions and behavior. Zhao et al. used a post-menopausal mouse model to verify the hypothesis that mitochondrial damage may contribute to cognitive impairment associated with estrogen deficiency, while Zhang S. et al. conducted a cross-sectional study in early menopausal women to evaluate estradiol-related structural changes in the brain. As suggested by De Filippi et al., Lima et al., Zanolie et al., Qin et al., and Palamarchuk and Vaillancourt, ovarian hormones and menstrual cycle modulate whole-brain turbulent dynamics, contribute to the sex differences in the reserpine-induced progressive animal model of PD, and modulate neural activity and pathways related to stressor detection and coping. Dai et al. reports clinical manifestations of variants of the DDX3X gene, which is associated with intellectual disability mostly in females (and only rarely in males).

Several papers contribute to advancing our understanding of schizophrenia. Rootes-Murdy, Zendehrough et al. and Jensen et al. discuss MRI data analysis methods to describe the structural and cognitive differences of schizophrenia patients and healthy controls. Rootes-Murdy, Goldsmith et al. provide an overview of neural changes and clinical presentations associated with delusions, a hallmark of certain psychotic disorders and neurodegenerative diseases. Liu et al. explore if metabolites of phospolipids may be used as biomarkers for therapeutic response in schizophrenia patients, while Bermperidis et al. use the human transcriptome to more generally differentiate between neuropsychiatric and neurological disorders as human embryonic stem cells become neurons. Cognitive functions in conditions of nutrient deprivation, high stress (associated to maternal post-partum and childhood bullying victimization), neurodegenerative disorders or Small Vessel Disease were addressed by Zhang Q. et al., Palamarchuk and Vaillancourt, Vandenbroucke et al., Gronewold and Engels, Liao et al., and Qin et al. Moreover, Shi et al. demonstrated that berberine treatment significantly restored cognitive impairment in sepsis mice, while Metaxas focused on the molecular interactions characterizing the pathogenesis of Alzheimer's Disease in a *C. elegans* model.

Finally, Saveko et al. and Nosikova et al. report on how motor function may be affected by extreme conditions, such as dry immersion and altered gravity situations in long term space missions, while Putman et al. investigated the effects of Galvanic vestibular stimulation in functional mobility tasks, to be potentially exploited by as astronauts, firefighters, high performance athletes, and soldiers.

The breadth and quality of the papers in this Research Topic provides important ground for future research and will hopefully serve as an inspiration for young neuroscientists. The Research Topic recognizes the breadth of scientific ideas and findings within the field, and given the cross-disciplinary nature of neuroscience, puts on record women's contribution to the scientific effort at large.

## Author contributions

All authors listed have made a substantial, direct, and intellectual contribution to the work and approved it for publication.

## Conflict of interest

The authors declare that the research was conducted in the absence of any commercial or financial relationships that could be construed as a potential conflict of interest.

## Publisher's note

All claims expressed in this article are solely those of the authors and do not necessarily represent those of their affiliated organizations, or those of the publisher, the editors and the reviewers. Any product that may be evaluated in this article, or claim that may be made by its manufacturer, is not guaranteed or endorsed by the publisher.

